# Clinical evaluation of fully automated thigh muscle and adipose tissue segmentation using a U-Net deep learning architecture in context of osteoarthritic knee pain

**DOI:** 10.1007/s10334-019-00816-5

**Published:** 2019-12-23

**Authors:** Jana Kemnitz, Christian F. Baumgartner, Felix Eckstein, Akshay Chaudhari, Anja Ruhdorfer, Wolfgang Wirth, Sebastian K. Eder, Ender Konukoglu

**Affiliations:** 1 Department of Imaging and Functional Musculoskeletal Research, Institute of Anatomy, Paracelsus Medical University, Strubergasse 21, 5020 Salzburg, Austria; 2 Chondrometrics GmbH, Ainring, Germany; 3 University of Vienna, Vienna, Austria; 4 ETH, Zurich, Switzerland; 5 Stanford University, Stanford, CA USA; 6 St. Anna Children’s Hospital, Vienna, Austria

**Keywords:** Muscle, Magnetic resonance imaging, Deep learning, Convolutional neural networks, Automated segmentation

## Abstract

**Objective:**

Segmentation of thigh muscle and adipose tissue is important for the understanding of musculoskeletal diseases such as osteoarthritis. Therefore, the purpose of this work is (a) to evaluate whether a fully automated approach provides accurate segmentation of muscles and adipose tissue cross-sectional areas (CSA) compared with manual segmentation and (b) to evaluate the validity of this method based on a previous clinical study.

**Materials and methods:**

The segmentation method is based on U-Net architecture trained on 250 manually segmented thighs from the Osteoarthritis Initiative (OAI). The clinical evaluation is performed on a hold-out test set bilateral thighs of 48 subjects with unilateral knee pain.

**Results:**

The segmentation time of the method is < 1 s and demonstrated high agreement with the manual method (dice similarity coeffcient: 0.96 ± 0.01). In the clinical study, the automated method shows that similar to manual segmentation (− 5.7 ± 7.9%, *p* < 0.001, effect size: 0.69), painful knees display significantly lower quadriceps CSAs than contralateral painless knees (− 5.6 ± 7.6%, *p* < 0.001, effect size: 0.73).

**Discussion:**

Automated segmentation of thigh muscle and adipose tissues has high agreement with manual segmentations and can replicate the effect size seen in a clinical study on osteoarthritic pain.

## Introduction

Thigh muscle deficits [1, 2] and accumulation of (local) adipose tissue [3–5] are important pathophysiological events in the context of the clinical science and management of musculoskeletal diseases such as knee osteoarthritis (OA) [1]. Muscles play an essential role in stabilizing the joints [1, 6], while excessive adipose tissue may induce a chronic inflammatory state by producing adipokines and inflammatory cytokines. Both are suggested to be involved in cartilage degradation, synovial inflammation, and bone erosion [3, 4]. Magnetic resonance imaging (MRI)-based analysis is increasingly used to study the association between thigh muscle and adipose tissue composition with knee OA [7–12]. Further, it has permitted to investigate the impact of training interventions on thigh tissue composition [13], as well as on functional and clinical outcomes of knee OA [5, 14]. Yet, evaluation of thigh muscle morphology and adipose tissue composition requires image segmentation, with the time needed for manual segmentations of thigh muscle and adipose tissue cross-sectional areas (CSAs), precluding the analysis of large databases and image repositories such as the Osteoarthritis Initiative (OAI) [15].

**Fig. 1:**
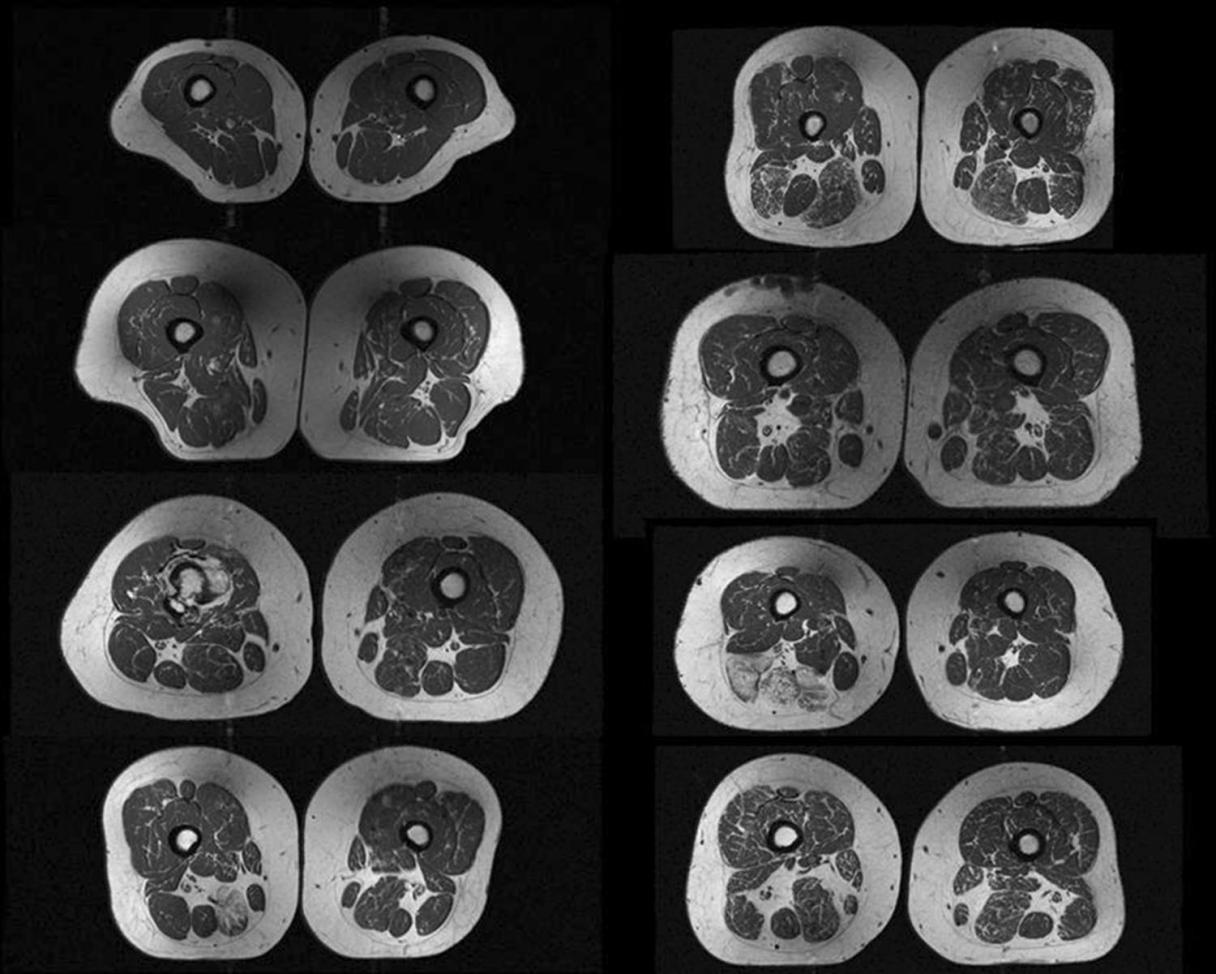
Thigh MRI s from eight OAI participants illustrating the intrasubject variability of thigh muscle and adipose tissue morphology, as well as intensity distortions

There exist several semi-automated [16–21] and fully automated [22–25] tools for thigh tissue volume and CSA segmentation to overcome the challenges in capturing the complex morphology and texture of thigh muscle and adipose tissue that are complicated by considerable inter-subject variability (Fig. 1) and potentially also artefacts as intensity distortions. Imperfections in MRI systems and interactions between the imaged subject and the electromagnetic field cause the sensitivity and hence, the image intensity scale to vary over the image.

Therefore, in previous published studies mainly continuous methods [18, 19, 24, 26, 27] and/or applied intensity inhomogeneity correction prior to segmentation were applied [19, 21, 22, 24]. Several of the discrete methods used k-means [19, 27], fuzzy c-means clustering [24, 28] for adipose tissue classification and focused on atlas-based segmentation methods for the segmentation [18, 24, 25, 27] of individual muscle heads or lean muscle tissue from whole MRIs. Similar segmentation techniques have also been applied to other body tissues [29–31]. With more data becoming available and recent advances in machine learning and computing infrastructure, segmentation techniques based on deep convolutional neural networks (CNN) are emerging as the new state-of-the-art [32, 33]. For this reason, CNNs are recently examined for musculoskeletal tissue segmentation of the knee joint [34–36] and thigh muscle MRIs by Ahmad et al. [37] and our group [38]. Ahmad et al. explored five pre-trained fully convolutional networks (FCN) with initiated weights for transfer learning for the quadriceps muscle (including the femoral bone and the medulla). The authors reached high dice similarity coefficients (DSC) of 0.95. While in the above paper [37], the FCN-8s showed the most accurate results, a modified 2D U-Net architecture achieved an ever better performance, when applied to segmentation of cardiac MRI data [39]. While, in general, these CNN methods show great potential for thigh muscle and adipose tissue segmentation, particularly in large clinical image repositories; it is important to demonstrate whether clinical observation can be reproduced using segmentations generated and thus to “clinically” validate the methodology developed. More specifically, our study aims to close this important gap between medical imaging innovation and its clinical application by reproducing a clinical effect observed in a previous published study and by comparing effect sizes. A fully automated segmentation method that is clinically validated will enable segmentation of large imaging repositories such as the OAI, where the MRIs of several thousand patients can be analyzed and used for the development of imaging biomarkers and ultimately the resulting diagnosis and treatment of diseases.

The aim of the current study was therefore: (i) to determine the agreement between a fully automated thigh muscle and adipose tissue segmentation method based on a 2D U-Net technique vs. manual segmentation and (ii) to test whether a previously published clinical study can be reproduced using the CNN algorithm [40] that has shown that patients display lower quadriceps CSAs in limbs with frequently painful knees compared with pain-free contralateral limbs. The latter “clinical evaluation” is particularly important to show the value of the fully automated algorithm for detecting small differences between groups and within subjects over time.

## Material and methods

### U-Net architecture

Convolutional neural networks are a type of multilayered artificial neural networks that can be used to analyze imaging data. The U-Net architecture is built upon the fully convolutional network (FCN), which is built only from successive locally connected convolution, and pooling layers, and a final upsampling layer. In contrast to FCNs, the U-Net (1) has symmetric downsampling and upsampling with deep skip connections and (2) the skip connections between the downsampling path and the upsampling path apply a concatenation operator instead of a sum. These skip connections intend to provide local spatial cues to the upsampling operator. Because of its symmetry, the network has a large number of feature maps in the upsampling path, which allows transferring information. Our proposed method also relies on data augmentation, adding random spatial transformations of existing data as additional training examples, to use the available annotated samples more efficiently and yield higher segmentation performance [32]. In this work, we used a modified 2D U-Net architecture, where the number of feature maps in the transpose convolutions of the upsampling path was set to the number of classes, which has been used previously for segmenting cardiac tissue [39]. We trained the architecture to optimize pixel-wise multi-class, where each pixel *i* in image X is assigned to a label $y_{i}\, { \in }\,L_{a} = \{ l_{o} , \ldots ,l_{L} \}$ and $p$ denotes the ground-truth probability distribution, and $q$ denotes the networks softmax output (Eq. 1) with mini-batch stochastic gradient descent using the ADAM optimizer [41] with a learning rate of 0.01. The network was trained on Nvidia Titan Xp GPU for 24 h.

1 $$C = \mathop \sum \limits_{i} m_{i} \mathop \sum \limits_{{l{ \in }L_{a} }} p\left( {y_{i} = l} \right)\log q(y_{i} = l|X).$$

### U-Net training

**Fig. 2:**
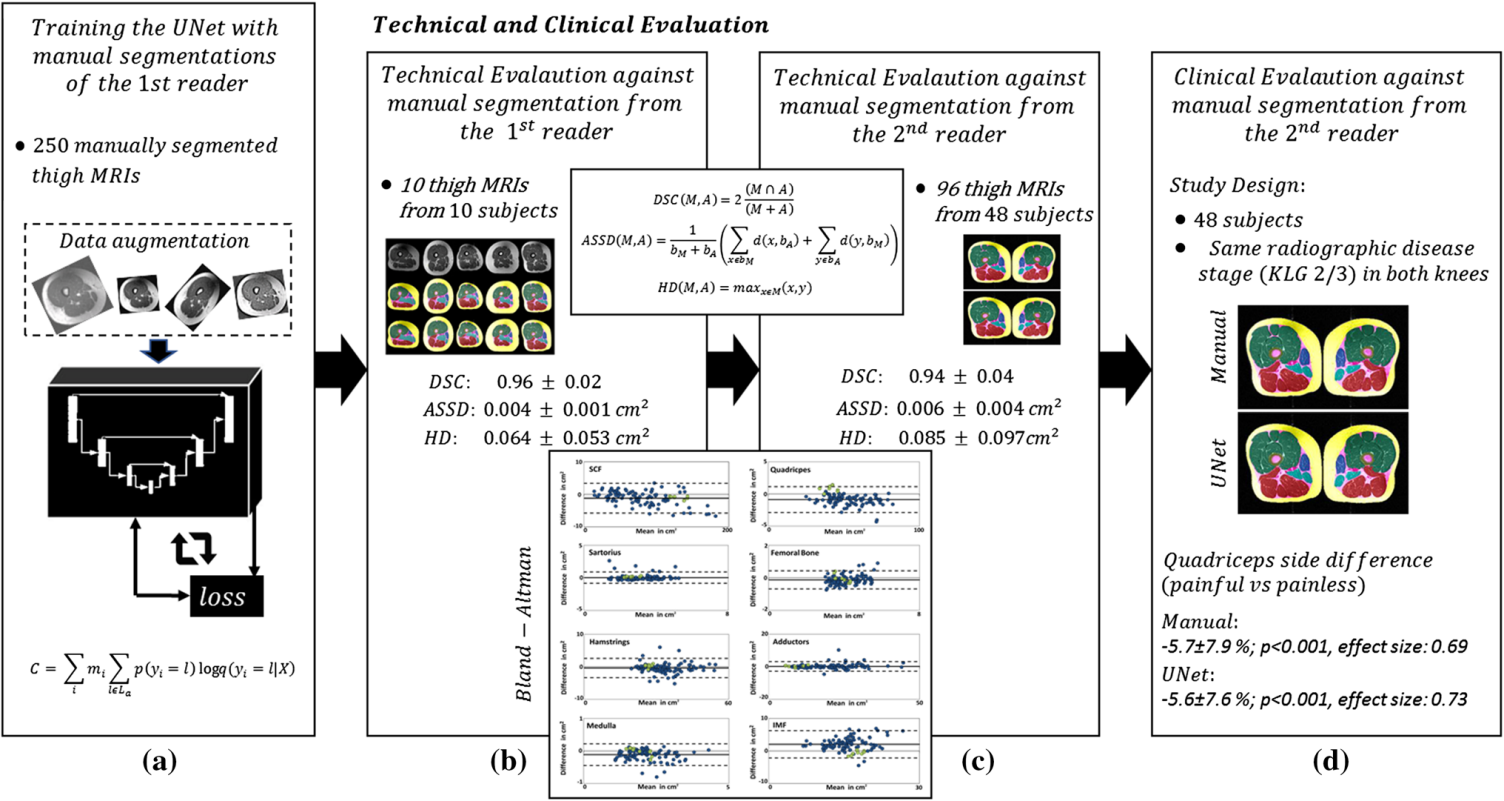
Graphical abstract and method overview: **a** the network was trained with a set of images and corresponding manual segmentations made by one reader (1st reader); **b** technical evaluation was performed using another dataset with corresponding manual segmentations made by the same reader (1st reader); **c** technical evaluation was repeated using 96 manual segmentations of another reader (2nd reader) who were manually acquired in the clinical study with 48 patients under **d** clinical evaluation was performed in comparison with data previously generated by the 2nd reader

In principal, the following steps were performed (Fig. 2):

- The network was trained with a set of images and corresponding manual segmentations made by one reader (1st reader)
- Technical evaluation was performed using another dataset with corresponding manual segmentations made by the same reader (1st reader)
- Technical evaluation was repeated using 96 manual segmentations of another reader (2nd reader) who were manually acquired in the clinical study with 48 patients under d)
- Clinical evaluation was performed in comparison with data previously generated by the 2nd reader.

#### Ad (a) training

The training set consisted of axial MR images from 222 participants (250 thighs: 202 left; 48 right) of OAI participants (male: 44%, age 65.5 ± 10.1 years, BMI 28.8 ± 4.8 kg/m^2^). In these, various muscle groups (i.e. quadriceps, hamstrings, adductors and sartorius), adipose tissue (i.e. subcutaneous fat [SCF], intermusclar fat [IMF]), and the femoral bone (including the cortex and the medulla) had been manually segmented to study the impact of pain [7, 8] and radiographic disease stage [42] on thigh muscle. The MR images had been acquired using a T1-weighted spin echo MRI sequence from the OAI (slice thickness 5 mm; in-plane resolution 0.98 mm; no inter-slice gap, repetition time 500 ms, echo time 10 ms) [15, 43] using a 3-T scanner (Siemens Trio, Siemens AG, Erlangen, Germany). Image acquisition encompassed 15 slices at a fixed distance from the distal femoral metaphysis [32]. Segmentation was performed at a single slice located at 33% of the femoral bone (from distal to proximal), the anatomical location that was consistently covered in all cases according to previously established criteria [44]. All MRI datasets were manually segmented by one reader (1st reader). Since both thighs are almost left–right symmetric, the right and left ones were mirrored to increase the number of training samples and randomly divided into a training set (*N* = 225 lefts and *N* = 225 right thighs) used to adjust the U-Net weights and into a validation set (*N* = 25 right and *N* = 25 left thighs) used to determine when to stop the training to avoid overfitting. Note that the validation set here is not used to evaluations, but only to determine when to stop the training.

#### Ad (b) technical evaluation with data from the same reader

The trained U-Net segmentation method was first applied to ten previously manual-segmented MRI datasets from the OAI segmented by the same reader (1st reader) that were not part of the training and validation set.

To determine the agreement between manual (M) and the fully automated (A) technique, the segmentations were compared using three different metrics. First, the dice similarity coefficient (DSC) was determined to measure the pixel overlap between A and M, normalized to their respective size (Eq. 2). The DSC is commonly used to evaluate the agreement between segmentation methods and relates the overlap of the segmentations to the total area of the segmentations. It takes pixel misclassification more strongly into account in smaller areas compared to larger ones. Therefore the average symmetric surface distance (ASSD) and Haussdorf distance (HD) were also included: The ASSD was determined as an indicator of the average segmentation error by the average of all the distances from each pixel on the boundary $b_{M}$ of M to the boundary $b_{A}$ of A and vice versa (Eq. 3). Finally, we determined the HD as an indicator of the largest segmentation error measuring the maximal distance from a pixel in A to a nearest pixel in M (Eq. 4):

2 $$DSC\left( {M,A} \right) = 2\frac{{\left( {M \cap A} \right)}}{{\left( {M + A} \right)}}.$$

3 $$ASSD\left( {M,A} \right) = \frac{1}{{b_{M} + b_{A} }}\left( {\mathop \sum \limits_{{x{ \in }b_{M} }} d\left( {x,b_{A} } \right) + \mathop \sum \limits_{{y{ \in }b_{A} }} d\left( {y,b_{M} } \right)} \right).$$

4 $$HD\left( {M,A} \right) = max_{{x{ \in }M}} \left( {x,y} \right).$$

#### Ad (c) technical evaluation with data from another reader (2nd reader)

As interobserver differences have been previously reported for thigh muscle and adipose tissue segmentations between different readers [45], this step was used to elucidate how technical evaluation parameters differ when the neural network trained by one reader is applied to data from another reader. To this end, the algorithm was applied to 48 different MRI from the OAI dataset, which have been manually segmented by another reader (2nd reader) who was involved in the clinical study described under (d). The same measures of similarity were used as described under (b).

The differences between the manual segmentations by the 2nd reader and the fully automated results of the pain study were examined using Bland–Altman analyses.

#### Ad (d) clinical evaluation

Finally, the trained U-Net segmentation method was applied to 48 patients from a previous published pain study segmented by the 2nd reader [40]. This study had aimed to determine whether thigh muscles differ in subjects with unilateral pain, i.e. between limbs with frequent knee pain (for at least one month during the past 12 months) compared with contralateral limbs without any knee pain over the past 12 months. These 48 subjects (31 women; 17 men; age 45–78 years) had been drawn from 4796 OAI participants, in whom both knees displayed the same radiographic stage osteoarthritis according to Kellgren and Lawrence grade (KLG) system for classification of osteoarthritis of knee and had been classified to be either bilaterally KLG2 or KLG3 [40]. Twenty-one participants displayed KLG2 (6 men, 15 women) and 27 bilateral KLG3 (11 men, 16 women) in both knees. Axial MR images were used to determine quadriceps, hamstrings, and adductors at 33% femoral length (distal to proximal).

As in the previously published paper [40], side differences between knees were determined and the standard deviation of these side differences was calculated. Paired t-tests were used to determine whether significant side differences in the quadriceps, hamstrings, and adductors appeared, with the effect size of significant differences being described using Cohen's D.

All statistical analyses were performed using SPSS version 24 (IBM Corp., USA) and Python 3.4 (Python Software Foundation, Delaware, United States).

## Results

**Fig. 3:**
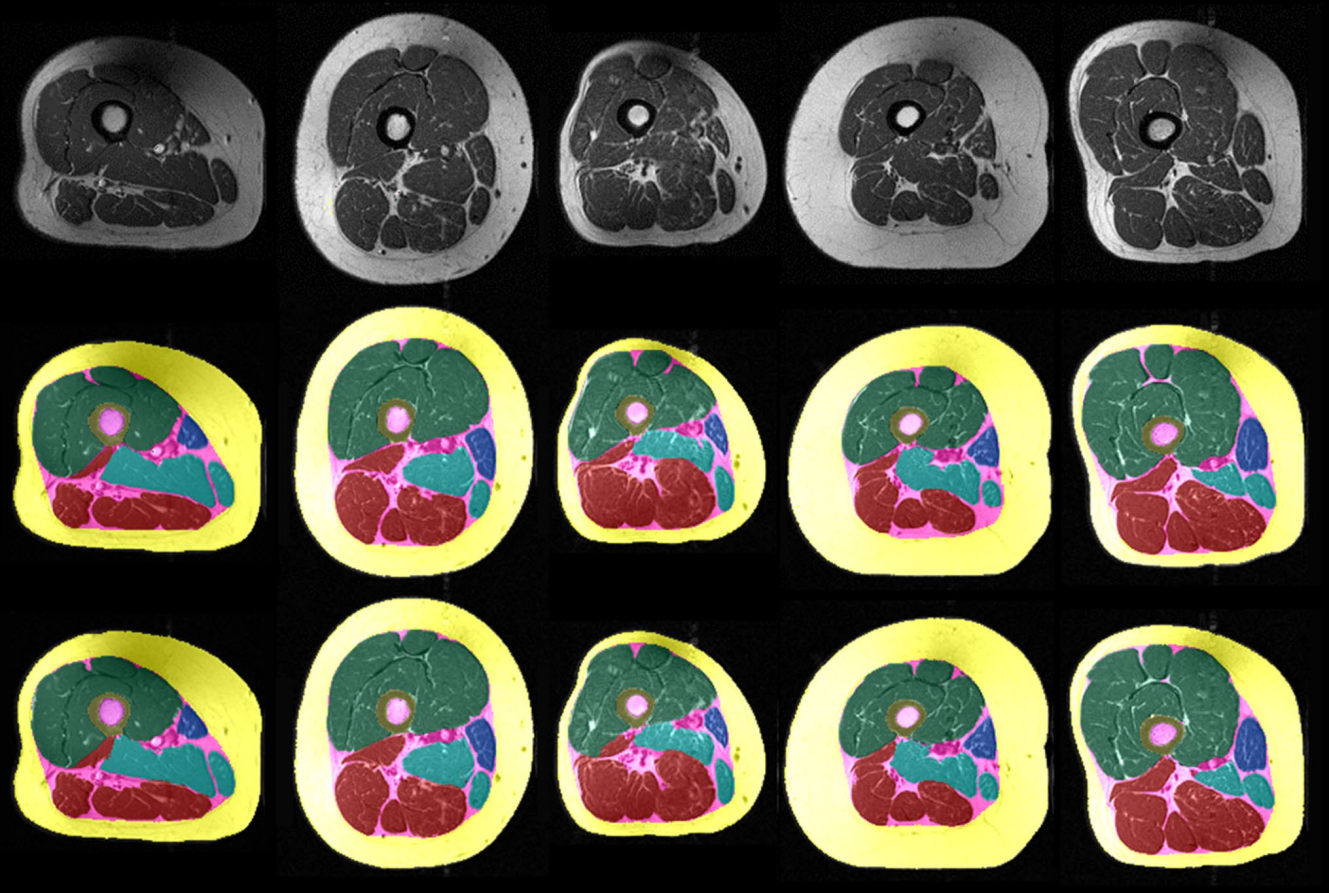
Example thigh MRI (33% distal–proximal) segmentation results from five OAI participants: original image (upper); manual segmentation results (middle); U-Net segmentation results (lower)

Figure 3 shows examples of different segmentations and some of the variability observed in the training dataset.

The agreement between manual and U-Net segmentation on the technical evaluation set (segmented by the same readers who’s segmentations had been used in the training and testing of the U-Net) was consistently high for all segmented structures (overall DSC (mean ± SD): 0.96 ± 0.02, overall ASSD: 0.004 ± 0.001, overall HD: 0.022 ± 0.001, Table 1). The DSC agreement was particularly high for the SCF, the quadriceps, the hamstrings, the femoral bone circumference, and the sartorius, and somewhat lower for femoral medulla, adductors, and IMF (Table 1).

**Table 1:** Agreement between manual and fully automated U-Net segmentation in the technical evaluation set; manual segmentations were acquired by the same reader (1st reader)

	DSC	ASSD	HD
SCF	0.99 ± 0.00	0.002 ± 0.001	0.067 ± 0.066
Quadriceps	0.98 ± 0.00	0.005 ± 0.001	0.082 ± 0.071
Flexors	0.98 ± 0.01	0.004 ± 0.001	0.075 ± 0.031
Adductors	0.91 ± 0.06	0.005 ± 0.002	0.058 ± 0.020
Sartorius	0.97 ± 0.01	0.004 ± 0.001	0.023 ± 0.013
Medulla	0.95 ± 0.02	0.004 ± 0.003	0.067 ± 0.143
Femoral bone	0.98 ± 0.02	0.002 ± 0.002	0.020 ± 0.022
IMF	0.90 ± 0.02	0.005 ± 0.001	0.116 ± 0.059
Overall	0.96 ± 0.02	0.004 ± 0.001	0.022 ± 0.032

Accuracy measured (mean ± SD) with dice similarity coefficient (DSC), average symmetric surface distance (ASSD), and Hausdorff distance; distances measured in cm^2^

The agreement between manual and U-Net segmentation, using training and evaluation data from different readers was also very high with an overall DSC of 0.94 ± 0.04 and overall ASS of 0.006 ± 0.004 cm^2^ and an overall HD of 0.085 ± 0.097 cm^2^ (Table 2). Yet, the measures of similarity were slightly lower than for the technical evaluation obtained from data from the same reader (Table 1).

**Table 2:** Agreement between manual and fully automated U-Net segmentation in the technical evaluation set; manual segmentations were acquired by another reader (2nd reader)

	DSC	ASSD	HD
SCF	0.97 ± 0.02	0.004 ± 0.002	0.057 ± 0.087
Quadriceps	0.98 ± 0.01	0.008 ± 0.006	0.109 ± 0.129
Flexors	0.96 ± 0.02	0.008 ± 0.004	0.110 ± 0.086
Adductors	0.93 ± 0.04	0.009 ± 0.006	0.101 ± 0.101
Sartorius	0.94 ± 0.09	0.006 ± 0.006	0.082 ± 0.171
Medulla	0.93 ± 0.03	0.004 ± 0.002	0.052 ± 0.127
Femoral bone	0.96 ± 0.04	0.003 ± 0.003	0.022 ± 0.028
IMF	0.80 ± 0.05	0.009 ± 0.002	0.150 ± 0.044
Overall	0.94 ± 0.04	0.006 ± 0.004	0.085 ± 0.097

Accuracy measured (mean ± SD) with dice similarity coefficient (DSC), average symmetric surface distance (ASSD), and Hausdorff distance; distances measured in cm^2^

**Fig. 4:**
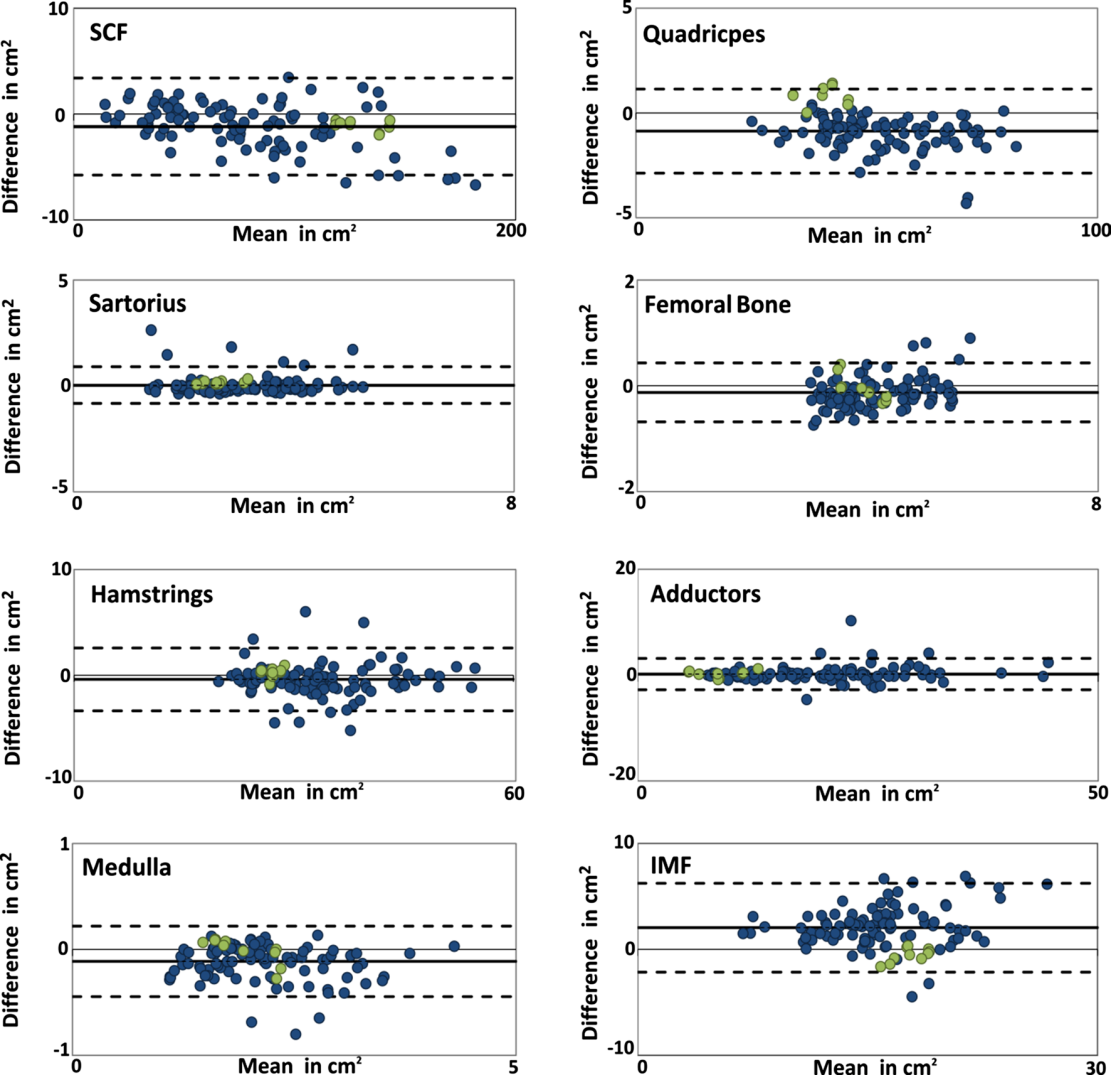
Bland–Altman plots showing the mean difference in cm^2^ between the manual and the fully automated U-Net segmentation results from the pain study (segmented by the 1st reader: green, segmented by the 2nd reader: blue). The limit of agreement (1.96 SD) is shown using dashed lines

In the technical evaluation (Fig. 4), the Bland–Altman analysis applied to data from the 1st reader showed a high agreement between the automated and manual segmentation results with a difference (absolute values in cm^2^ (percent values relative to size of structure)) of − 1.1 cm^2^ (− 0.8%) for the SCF, + 0.8 cm^2^ (+ 2.1%) for the quadriceps, + 0.3 cm2 (− 1.0%) for the hamstring, + 0.2 cm^2^ (+ 1.5%) for the adductors, and + 0.02 cm^2^ (+ 0.7%) for the Sartorius and -0.5 cm2 (− 3.0%) IMF CSAs (Fig. 4). When applied to data from the 2nd reader, the Bland–Altman analysis showed good agreement between the automated and manual segmentation results with a difference (absolute values in cm^2^ ( percent values relative to size of structure) of − 1.2 cm^2^ (1.5%) for the SCF, − 1.0 cm^2^ (− 2.0%) for the quadriceps, − 0.45 cm^2^ (− 1.3%) for the hamstring,  + 0.2 cm^2^ (+ 1.5%) for the adductors, and + 0.02 cm^2^ (+ 0.7%) for the Sartorius CSAs (Fig. 4). Some systematic deviations between the automated and the manual segmentation methods were observed with + 2.3 cm^2^ (+ 14.6%) for the IMF CSAs (Fig. 4).

**Fig. 5:**
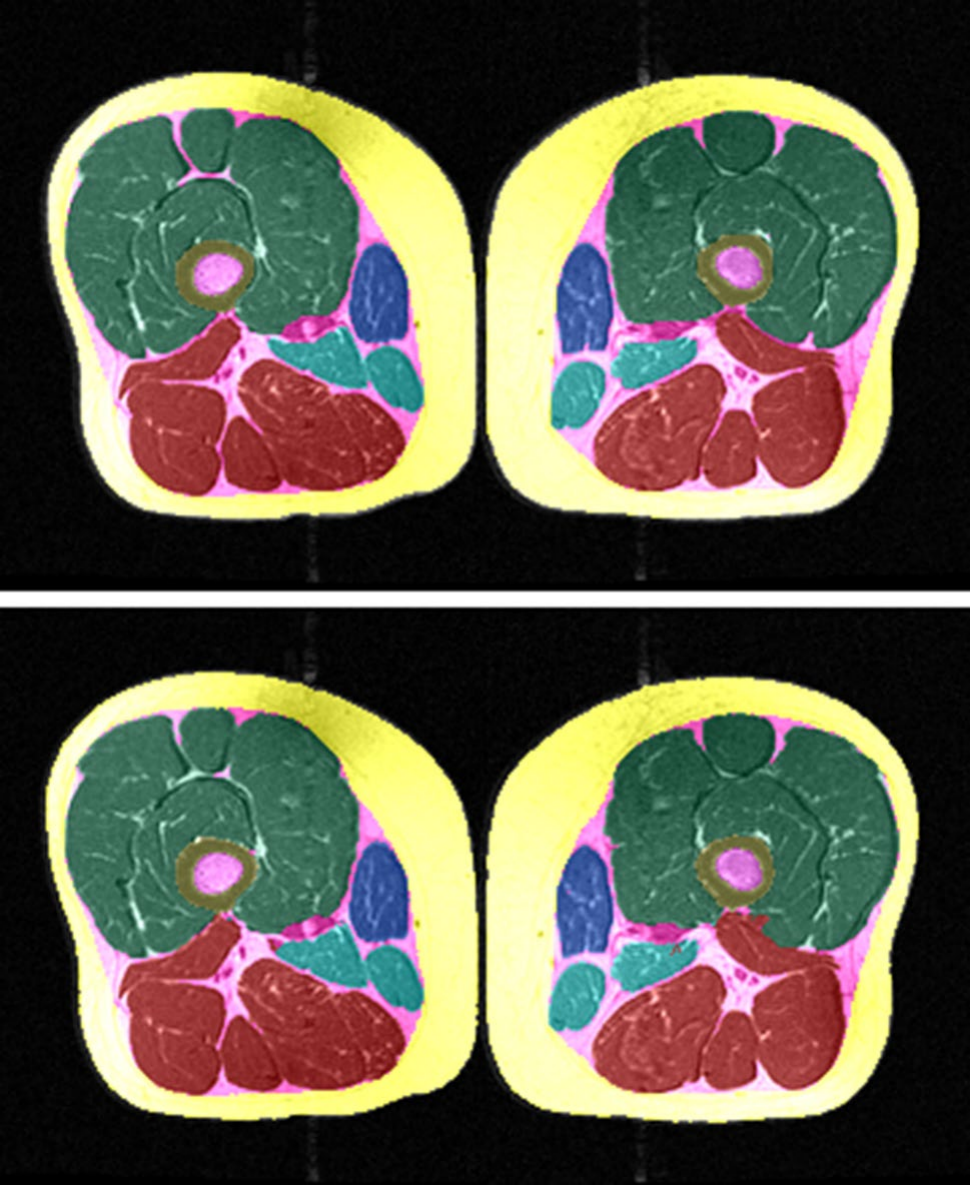
Side differences using manual und U-Net segmentation techniques of thigh MRI (33% distal–proximal) in bilateral knees with the same radiographic disease stage, but unilateral frequent pain; painful knee (right side); painless knee (left side); manual segmentation (upper) and U-Net segmentation (lower)

In the clinical evaluation study (Fig. 5), painful knees displayed significantly lower quadriceps CSAs for analyses performed with both segmentation methods (manual:  − 5.7 ± 7.9%, *p* < 0.001, effect size: 0.69; fully automated: − 5.6 ± 7.6%, *p* < 0.001, effect size: 0.73) than painless contralateral knees (Table 3). The CSAs of the hamstrings and adductors, in contrast, did not show any significant side differences using either segmentation method (Table 3). No statistically significant differences were observed for the other muscle groups, the SCF or the IMF between both segmentation techniques, but as can be appreciated by Table 3, the mean values and standard deviations on either side were very similar between both methods. Values of IMF CSAs obtained from the automated method tended, however, to be somewhat (approx. 15%) smaller than the CSAs obtained from manual segmentation (Table 3).

**Table 3:** Measured side differences in muscle and adipose tissue cross-sectional areas (CSA) between manual und U-Net segmentation techniques of thigh MRI (33% distal–proximal) in OAI participants with the same radiographic disease stage in both knees, but unilateral frequent pain; painful knee vs. painful knee

	Painful knee		Painless knee		Differences painful vs. painless		
	Mean	SD	Mean	SD	Mean%	SD%	*p *value
Manual							
Quadriceps	**50.79**	**12.26**	**53.79**	**13.45**	**− 5.73**	**7.92**	** < 0.001**
Hamstrings	33.27	7.97	33.67	7.93	− 1.21	8.03	0.292
Adductors	14.15	5.61	14.33	5.8	− 1.24	22.6	0.71
SCF	77.81	38.34	76.66	37.88	− 1.38	6.91	0.158
IMF	16.88	3.9	17.19	4.42	1.84	10.96	0.137
Automated							
Quadriceps	**51.83**	**12.32**	**54.83**	**13.72**	**− 5.63**	**7.64**	** < 0.001**
Hamstrings	33.76	7.93	34.09	7.92	− 0.98	6.56	0.299
Adductors	13.92	5.21	14.15	5.76	− 1.62	23.29	0.63
SCF	78.83	39.43	78.01	39.25	− 0.96	6.28	0.137
IMF	14.58	3.54	14.77	3.64	1.23	10.63	0.435

Bold signifies *p* < 0.001

## Discussion

The aim of the current study was to evaluate a rapid fully automated U-Net segmentation method for thigh muscle CSA segmentation from MRI that is suitable the analysis of large imaging databases in clinical trials. For this purpose, we evaluated the agreement between the fully automated segmentations and previously performed manual segmentations using data from the reader that performed the manual segmentations used to train the U-Net and using data from a second reader that were not part of the training or validation set. In a second step, the current U-Net segmentation method was able to reproduce the results from a previous clinical study, in which we had observed that the quadriceps of limbs with frequently painful knees shows lower CSAs compared with contralateral knees without knee pain.

The results from the current study showed high agreement (DSCs > 0.95) between the fully automated U-Net vs. manual segmentation approach for SCF, quadriceps, hamstrings, and femoral bone segmentations, independent of whether the algorithm was compared to the segmentations of the same or a different reader. The agreement for adductors, medulla, and sartorius was still high, but slightly lower (DSCs > 0.91) and in a similar range for both readers. The agreement for IMF was still good (DSCs > 0.90), when the U-Net was applied to segmentations from the same reader and was notably lower (DSCs > 0.80), when the U-Net was applied to segmentations from a different reader. This was consistent with the outcome of the Bland–Altman plots: the fully automated method showed a good agreement with the manual segmentations from both readers for most of the structures. Only the measurement of the IMF CSAs showed a considerable bias, when the U-Net was applied to data from a different reader. As outlined previously, the method presented here is fully automated and not dependent on a specific reader. The difference between automated and manual segmentation observed here, however, is well in the range of the interobserver variability of manual segmentation reported in previous studies [45]. When applied to the data from the clinical study with unilateral pain subjects, the proposed fully automated algorithm detected similar side differences in quadriceps CSAs, but with substantially less time needed for the analysis (< 1 s) than for current (semi-) automated (3–6 min) or manual segmentation techniques (60–90 min) depending on the reader and the image quality.

Prescott et al. used a numerical analysis-based level set approach and reported DSCs of 0.69 ± 0.16 (vastus medialis) − 0.82 ± 0.08 (vastus lateralis) in the individual quadriceps heads [17]. Trotter et al. focused on the individual quadriceps heads as well, reaching a DSC of 0.87 ± 0.11 for the fully automated multi-atlas framework [18]. Baudin et al. reported an average DSC of 0.86 ± 0.07 for individual thigh muscle heads combining a statistical shape atlas with a random walks graph [26]. Andrews et al. presented a probabilistic shape model framework and reported a mean DSC of 0.81 ± 0.07 for the segmentation of all individual thigh muscle heads [23]. Yang et al. used a voxel classifier combined with morphological operations in four contrast Dixon MR images. The authors reached a DSC of 0.96 ± 0.03 for the SCF, 0.80 ± 0.03 for the IMF and 0.97 ± 0.01 for the combined thigh muscles [22]. Karlsson et al. based their work on a multi-atlas segmentation approach for the muscle tissue segmentation from the whole body and reached a true positive classification from 0.93 ± 0.01 to 0.93 ± 0.03 [24]. Orgiu et al. introduced a discrimination of muscle and adipose tissue from T1-weighted MRIs of the thigh using a fuzzy c-mean algorithm and morphologic operators reporting a mean sensitivity above 96%, mean relative area difference of 1.8%, 2.7%, and 2.5%, respectively [28]. A first attempt for quadriceps MRI segmentation based on deep neural networks was undertaken by Ahmad et al. [37]. The authors explored five pre-trained deep learning models FCN-AlexNet, FCN-32s, FCN-16s, FCN- 8s with initiated weights for transfer learning and PSPNet with two different optimizations as Stochastic Gradient Descent and ADAM for quadriceps (including femoral bone and medulla as one large segmentation label), where the FCN-8s showed combined with the ADAM with quick processing time for inferencing as the best all-around deep learning model with a DSC of 0.95 for the quadriceps and processing time of 0.117s per image. In our study, we obtained an average DSC of 0.98 for the quadriceps, both when using evaluation data segmented by the same or by another reader (in relation to the training dataset) and hence, were able to improve upon this previous approach. Also, we included other and far more complex thigh MRI structures, such as the other muscle groups and IMF, and reached an overall DSC of 0.96 for an evaluation dataset from the same reader and an overall DSC of 0.94 for that from the a different reader, improving upon current state of the art.

More importantly, in the previous approaches [17, 18, 22-24, 26, 28, 37] the performance was not evaluated in the setting of a clinical study. The ability to reproduce relatively small side differences in the quadriceps muscle shown previously is promising for the application of the automated method in future studies, in particular for muscle and adipose tissue of the thigh that are in focus in knee OA [2, 13].

A potential limitation of the study is that the proposed fully automated segmentation method was trained only for a particular anatomical location (33% level of the femoral bone: distal–proximal) and not for other CSAs or a volumetric analysis. However, muscle CSAs acquired at the 33% level were shown to be strongly correlated with 3D muscle volume [46] and found to be sensitive to longitudinal change or cross-sectional differences in several clinical studies [12, 47, 48]. In addition, a longitudinal reduction of CSAs acquired at 33% of the femoral length was shown to be associated with muscle strength loss in patients with concurrent increase in KOA pain [10].

Another potential limitation is that the fully automated method showed a bias toward manual segmentation for the IMF that was greater when the method was applied to data from a 2nd reader (14.6%, DSC: 0.80), whose segmentations were not part of the training and validation set, than when applied to data from the 1st reader (− 3.0%, DSC: 0.90) whose data were used to train the U-Net. This difference between two readers is consistent with results from previous studies that reported an interobserver variability between manual segmentations of two different readers of 18.4/ 14.7% for the IMF, before/after quality control, respectively [45]. Yet, the DSC observed for IMF using data from the same reader compares quite favorable to the literature, while the DSC achieved with data from the 2nd reader is still comparable with the best achieved results of 0.80 in a study of Yang et al. using DIXON MRIs [22]. Further, since the observed effect was systematic and therefore similar for all patients, measuring (side) differences or longitudinal change in the IMF may not be strongly affected. Yet, future studies will have to establish the sensitivity to change for IMF and SCF, for instance during weight gain or loss.

The strength of the current study was that it not only assessed the agreement between manual and automated segmentation, but it also showed that the results of a clinical study could be reproduced using this new method.

## Conclusion

Our novel approach of muscle segmentation based on a U-Net is shown to be accurate and can thus be applied to fully automated evaluation of large datasets considerably faster (< 1 s) than for current (semi-) automated (3–6 min) or manual segmentation techniques (60–90 min). More importantly, the effect shown in a clinical study that knees with unilateral frequent pain demonstrate lower CSAs of the quadriceps (but not of other thigh muscles) compared with contralateral knees without knee pain was reproduced and showed a comparable effect size to that of manual segmentation.
